# Deer antler extract potentially facilitates xiphoid cartilage growth and regeneration and prevents inflammatory susceptibility by regulating multiple functional genes

**DOI:** 10.1186/s13018-021-02350-4

**Published:** 2021-03-22

**Authors:** Mengqi Guan, Daian Pan, Mei Zhang, Xiangyang Leng, Baojin Yao

**Affiliations:** 1 College of Traditional Chinese Medicine, Changchun University of Chinese Medicine, Changchun, 130117 China; 2 Jilin Ginseng Academy, Changchun University of Chinese Medicine, Changchun, 130117 China; 3 Innovation Practice Center, Changchun University of Chinese Medicine, Changchun, Jilin, 130117 China

**Keywords:** Deer antler extract, Xiphoid cartilage, RNA sequencing, Molecular regulation, Growth, Regeneration

## Abstract

**Background:**

Deer antler is a zoological exception due to its fantastic characteristics, including amazing growth rate and repeatable regeneration. Deer antler has been used as a key ingredient in traditional Chinese medicine relating to kidney and bone health for centuries. The aim of this study was to dissect the molecular regulation of deer antler extract (DAE) on xiphoid cartilage (XC).

**Methods:**

The DAE used in this experiment was same as the one that was prepared as previously described. The specific pathogen-free (SPF) grade Sprague-Dawley (SD) rats were randomly divided into blank group (*n* =10) and DAE group (*n* =10) after 1-week adaptive feeding. The DAE used in this experiment was same as the one that was prepared as previously described. The rats in DAE group were fed with DAE for 3 weeks at a dose of 0.2 g/kg per day according to the body surface area normalization method, and the rats in blank group were fed with drinking water. Total RNA was extracted from XC located in the most distal edge of the sternum. Illumina RNA sequencing (RNA-seq) in combination with quantitative real-time polymerase chain reaction (qRT-PCR) validation assay was carried out to dissect the molecular regulation of DAE on XC.

**Results:**

We demonstrated that DAE significantly increased the expression levels of DEGs involved in cartilage growth and regeneration, but decreased the expression levels of DEGs involved in inflammation, and mildly increased the expression levels of DEGs involved in chondrogenesis and chondrocyte proliferation.

**Conclusions:**

Our findings suggest that DAE might serve as a complementary therapeutic regent for cartilage growth and regeneration to treat cartilage degenerative disease, such as osteoarthritis.

## Background

Xiphoid cartilage (XC), also known as xiphoid process, is a small cartilaginous region located in the lower part of the sternum [1]. The major function of XC is to serve as an attachment for soft tissues and helps protect the internal thoracic viscera, such as the heart and lungs [2]. Although the XC is hided in the infrasternal angle, it is still vulnerable to be broken, usually caused by a chest trauma, and results in chest or abdominal pain, termed xiphodynia or xiphoidalgia [3]. The major cause for the pain syndrome is due to the injury in the XC region accompanied with inflammation [4]. Since cartilage is solely composed of cells, namely, chondrocytes, which have very poor capacities for self-renewal, cartilage regeneration remains one of the major challenges in this century [5].

Deer antler is a zoological exception due to its fantastic characteristics, including amazing growth rate and repeatable regeneration [6]. The maximal growth rate during antler growth can reach to 2 cm per day, representing the fastest growth rate of tissue growth among mammalian species [7]. Antler growth is powered by the proliferation and differentiation of cells resided in the antler tip, known as antler growth center, which is a cartilaginous structure classified into different zones, such as mesenchyme, precartilage, cartilage, and mineralized cartilage, and the process of antler growth resembles that of endochondral ossification during long bone development [8, 9]. Deer antler has been used as a key ingredient in traditional Chinese medicine relating to kidney and bone health for centuries [10]. However, the regulation of deer antler on cartilage homeostasis and development remains largely unknown.

In recent years, our group has carried out a series of studies for the purpose of getting insight into the molecular control of deer antler on the regulation of chondrocytes. Our findings indicate that the major active components in deer antler are aqueous proteins, which account for 70% of the freshly prepared aqueous extract (DAE) [11]. Furthermore, DAE dramatically facilitate chondrocyte viability and hold chondrocytes in a continuously proliferative state, while prevent chondrocytes from further maturation, differentiation, and apoptosis [11, 12]. In the present study, we treated rats with DAE, and analyzed the gene expression profiles of xiphoid cartilage by using RNA sequencing (RNA-seq) technology in combination with quantitative real-time polymerase chain reaction (qRT-PCR) verification method to uncover the molecular control of DAE on cartilage regulation. We demonstrated that DAE significantly increased the expression levels of DEGs involved in cartilage growth and regeneration, but decreased the expression levels of DEGs involved in inflammation, and mildly increased the expression levels of DEGs involved in chondrogenesis and chondrocyte proliferation.

## Methods

### Experimental animals and treatment

The specific pathogen-free (SPF) grade Sprague-Dawley (SD) rats at the age of 7-week old were purchased from the Changchun Yisi Laboratory Animal Technology Co., Ltd. (Changchun, China) with the permission number SCXK (Ji) 2016-0003. The rats were housed in an air conditioned and light/dark (12/12 h) cycled room, with the temperature range from 23 to 25 °C in combination with a relative humidity of 50% and free access to food and water. The animal protocol was approved by the Institutional Animal Care and Use Committee of Changchun University of Chinese Medicine and all experimental procedures were performed in accordance with corresponding standards and guidelines. After 1-week adaptive feeding, the rats were randomly divided into two groups: blank group (*n* =10) and DAE group (*n* =10). The DAE used in this experiment was same as the one that was prepared as previously described [11], and the administration was carried out as previously described [13]. Briefly, the rats in DAE group were fed with DAE for 3 weeks at a dose of 0.2 g/kg per day according to the body surface area normalization method [14], and the rats in blank group were fed with drinking water.

### Tissue collection and RNA isolation

After 3-week DAE administration, all rats were killed by carbon dioxide euthanasia and cervical dislocation. Xiphoid cartilage from each rat was carefully removed from the most distal edge of the sternum with a scalpel blade. The samples from each group were pooled together separately, and grinded it to fine powders using a pestle and mortar with liquid nitrogen. Total RNA from each group was extracted from the cartilage tissues with the TRIzol reagent (Invitrogen, USA) in accordance with the company’s protocols. RNA quality was assessed using an Agilent 2100 Bioanalyzer (Agilent Technologies, USA).

### Library preparation and Illumina sequencing

Library construction was prepared with the TruSeq Stranded mRNA kit (Illumina, USA) in accordance with the manufacturer’s recommendations. Briefly, mRNA was purified, fragmented, and reverse transcribed into double-stranded cDNA followed by end repair and adapter ligation. The generated fragments were further selectively amplified by polymerase chain reaction (PCR) to generate the libraries. Transcriptome sequencing was performed on an Illumina HiSeq 2500 platform (Illumina, USA) with a paired-end read of 150 bp in length.

### Data analysis

The raw reads were filtered via perl scripts to yield high-quality clean reads by excluding the adapter sequences, eliminating the unknown nucleotides and removing the low-quality reads. The clean reads were aligned to the rat (*Rattus norvegicus*) genome via HISAT [15], and gene expression levels were quantified with the FPKM method [16]. Differentially expressed genes (DEGs) were detected using the DEGseq method [17]. Genes with a log_2_ fold change ≥1 or ≤−1 and a *p* value ≤0.001 were defined as significantly differentially expressed. Enrichment analysis was further carried out by mapping the DEGs into Gene ontology (GO) and Kyoto Encyclopedia of Genes and Genomes (KEGG) databases using an R function phyper accompanied with multiple testing corrections. GO terms or KEGG pathways with an adjusted *p* value (*Q* value) less than 0.05 were regarded as significantly enriched [18].

### Validation by qRT-PCR assay

Gene expression levels of selected DEGs from RNA-seq analysis were validated by qRT-PCR assay. Briefly, total RNA used for RNA-seq was used as a template for cDNA synthesis via the iScript cDNA Synthesis Kit (Bio-Rad, USA) and subsequently amplified on the CFX Connect Real-Time PCR Detection System (Bio-Rad, USA) by using the SsoAdvanced Universal SYBR® Green Supermix (Bio-Rad, USA) in accordance with the manufacturer’s instructions. The relative mRNA expression levels were calculated according to the 2^−ΔΔCT^ algorithm, and the rat glyceraldehyde 3-phosphate dehydrogenase gene (Gapdh) was applied as a reference gene for normalization [19].

## Results

### Statistics summary of transcriptome sequencing and assembly

All of the raw reads from xiphoid cartilage samples of rats with or without the treatment of DAE were deposited into the NCBI Sequence Read Archive (SRA) database with a BioProject accession number PRJNA611513. As shown in Table 1, after removing adaptors and low-quality reads, a total of 45,373,826 (blank group) and 41,652,368 (DAE group) clean reads were obtained in each of the profiles, respectively. The quality control results showed that the Q30 value was above 91%, and the GC content was around 52~53% in each group, indicating high accuracy and reliability of the RNA-seq data. Among the clean reads, approximately 41 million (blank group) and 38 million (DAE group) reads were mapped to the rat (Rattus norvegicus) genome, subsequently, 12,632 out of 15,275 (blank group) and 12,323 out of 14,970 (DAE group) known transcripts were identified by searching against the NCBI non-redundant (NR) protein database and Swiss-Prot database, respectively.

**Table 1:** Statistics summary of transcriptome sequencing and assembly

Statistics	Blank	DAE
Clean reads	45,373,826	41,652,368
Q30 percentage	91.17	91.60
GC percentage	51.94	52.90
Total mapped reads	41,077,322	37,894,242
Total transcripts	15,275	14,970
Known transcripts	12,632	12,323

### Differential expression and functional enrichment analysis

**Fig. 1:**
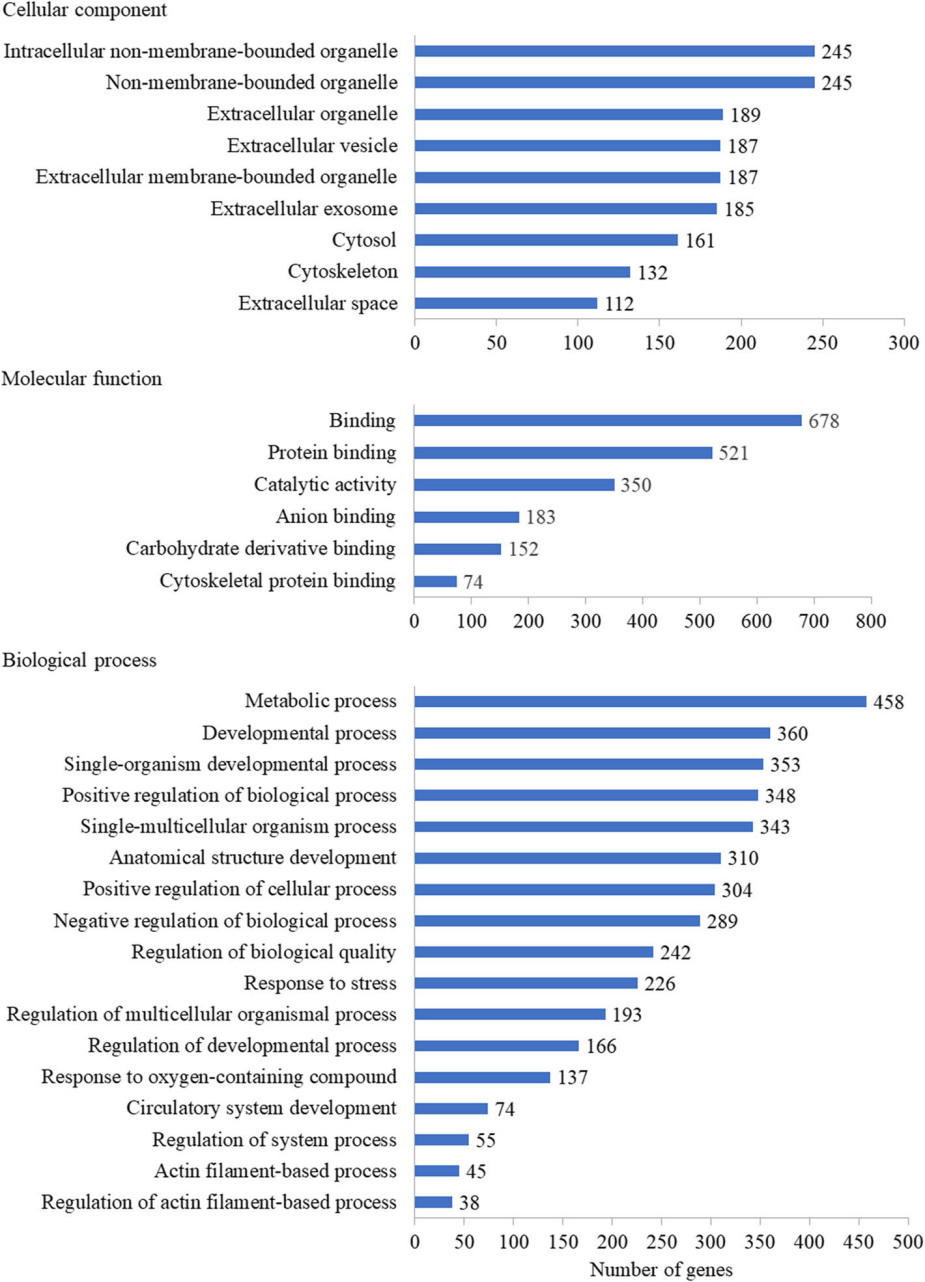
GO enrichment analysis of XC under DAE treatment. The *x*-axis indicates the number of mapped genes in a category, and the *x*-axis indicates the significantly enriched GO terms (*p* <0.05) in different categories including cellular component, molecular function, and biological process

**Fig. 2:**
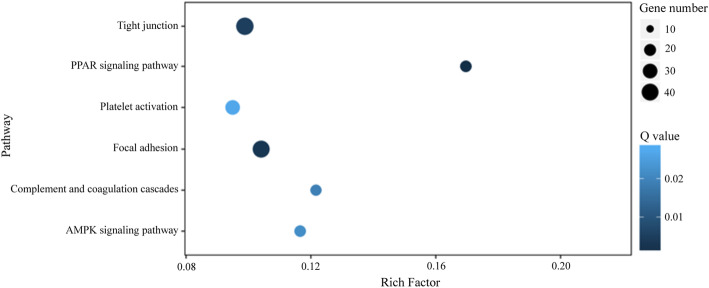
KEGG enrichment analysis of XC under DAE treatment. The *x*-axis indicates the rich factor that is presented by the ratio of DEG number in the total gene number of a defined pathway, and the *x*-axis indicates the name of enriched pathway. The color of the dots represents the range of the *Q* value, and the size of the dots represents the number of DEGs mapped to a defined pathway, respectively

In total, 892 DEGs were identified between the blank group and the DAE group, with 181 significantly upregulated genes and 711 significantly downregulated genes by comparing the blank group and DAE group based on the following criteria: log_2_ fold change ≥1 or ≤−1 and *p* ≤0.001, as shown in Table 2. GO enrichment analysis was performed to identify the biological importance of DEGs under DAE treatment, as shown in Fig. 1. In the cellular component category, the DEGs were predominantly located in the organelle, vesicle, and exosome regions. In the molecular function category, the DEGs were mainly involved in the binding and catalytic activities. In the biological process category, the DEGs primarily participated in the metabolic and developmental processes. KEGG pathway enrichment analysis was performed to further determine the biological pathways in which these DEGs may be participated, as shown in Fig. 2. The DEGs were predominantly mapped to the following pathways, including tight junction, PPAR, platelet activation, focal adhesion, complement and coagulation cascades, and AMPK.

**Table 2:** Statistic overview of DEGs (DAE vs. blank)

Statistics	Number
Differentially expressed mRNAs	892
Upregulated mRNAs	181
Downregulated mRNAs	711

### DAE remarkably promote the expression levels of DEGs participated in cartilage growth and regeneration

Among the significantly upregulated DEGs, 17 DEGs participated in cartilage growth and regeneration were pointed out, including connective tissue growth factor (Ctgf), fibronectin (Fn1), aggrecan core protein (Acan), lysyl oxidase homolog 4 (Loxl4), protein CYR61 (Cyr61), sestrin-3 (Sesn3), proteoglycan 4 (Prg4), filamin-B (Flnb), frizzled-7 (Fzd7), bone morphogenetic protein 7 (Bmp7), bone morphogenetic protein 6 (Bmp6), golgin subfamily B member 1 (Golgb1), peptidase inhibitor 15 (Pi15), krueppel-like factor 9 (Klf9), zinc finger protein GLI2 (Gli2), ephrin-A5 (Efna5), and zinc finger transcription factor Trps1(Trps1), as shown in Table 3.

**Table 3:** Comparison of gene expression patterns of DEGs participated in cartilage growth and regeneration

Gene name	Expression level (FPKM)		log_2_ fold change (DAE/blank)	*p* value
	Blank	DAE		
Connective tissue growth factor (Ctgf)	1071.72	2155.72	1.01	0
Fibronectin (Fn1)	703.09	1775.29	1.34	0
Aggrecan core protein (Acan)	694.33	1466.54	1.08	0
Lysyl oxidase homolog 4 (Loxl4)	88.25	191.04	1.11	0
Protein CYR61 (Cyr61)	29.43	60.45	1.04	3.52E−44
Sestrin-3 (Sesn3)	16.14	33.95	1.07	2.12E−39
Proteoglycan 4 (Prg4)	15.12	31.45	1.06	1.02E−49
Filamin-B (Flnb)	12.00	30.41	1.34	1.68E−151
Frizzled-7 (Fzd7)	6.71	14.64	1.13	1.47E−18
Bone morphogenetic protein 7 (Bmp7)	4.83	14.31	1.57	1.97E−25
Bone morphogenetic protein 6 (Bmp6)	6.80	13.61	1.00	2.85E−08
Golgin subfamily B member 1 (Golgb1)	4.44	10.82	1.29	4.13E−64
Peptidase inhibitor 15 (Pi15)	3.14	9.50	1.60	4.44E−10
Krueppel-like factor 9 (Klf9)	4.24	9.49	1.16	9.91E−13
Zinc finger protein GLI2 (Gli2)	4.02	8.50	1.08	1.48E−24
Ephrin-A5 (Efna5)	4.05	8.39	1.05	7.06E−11
Zinc finger transcription factor Trps1(Trps1)	1.56	3.72	1.25	1.98E−15

### DAE remarkably suppressed the expression levels of DEGs participated in inflammation

Among the significantly upregulated DEGs, 30 DEGs participated in inflammation were recognized, including 40S ribosomal protein S24 (Rps24), fatty acid-binding protein (Fabp4), beta-2-microglobulin (B2m), mimecan (Ogn), retinoid-binding protein 7 (Rbp7), lysozyme C-1 (Lyz1), adiponectin (Adipoq), CCAAT/enhancer-binding protein delta (Cebpd), thyroid hormone-inducible hepatic protein (Thrsp), DnaJ homolog subfamily C member 15 (Dnajc15), CCAAT/enhancer-binding protein beta (Cebpb), platelet glycoprotein 4 (Cd36), resistin (Retn), collagen alpha-1(IV) chain (Col4a1), and angiotensinogen (Agt), as shown in Table 4.

**Table 4:** Comparison of gene expression patterns of DEGs participated in inflammation

Gene name	Expression level (FPKM)		log_2_ fold change (DAE/blank)	*p* value
	Blank	DAE		
40S ribosomal protein S24 (Rps24)	4192.80	2010.43	−1.06	0
Fatty acid-binding protein (Fabp4)	2432.08	468.50	−2.38	0
Beta-2-microglobulin (B2m)	516.35	237.92	−1.12	0
Osteoglycin (Ogn)	140.89	67.67	−1.06	2.91E−46
Retinoid-binding protein 7 (Rbp7)	253.48	65.29	−1.96	4.24E−116
Lysozyme C-1 (Lyz1)	120.53	55.53	−1.12	6.30E−56
Adiponectin (Adipoq)	293.37	45.45	−2.69	0
CCAAT/enhancer-binding protein delta (Cebpd)	80.67	38.92	−1.05	5.26E−32
Thyroid hormone-inducible hepatic protein (Thrsp)	221.82	36.82	−2.59	0
DnaJ homolog subfamily C member 15 (Dnajc15)	70.79	34.30	−1.05	2.10E−18
CCAAT/enhancer-binding protein beta (Cebpb)	70.21	32.67	−1.10	5.94E−40
Platelet glycoprotein 4 (Cd36)	112.80	27.68	−2.03	4.07E−293
Resistin (Retn)	144.77	25.36	−2.51	2.83E−84
Collagen alpha-1(IV) chain (Col4a1)	43.97	18.93	−1.22	3.28E−137
Angiotensinogen (Agt)	63.79	18.67	−1.77	9.28E−99
O-acetyl-ADP-ribose deacetylase MACROD1 (Fragment) (Macrod1)	38.50	16.56	−1.22	6.04E−18
Interferon alpha-inducible protein 27-like protein 2B (Ifi27l2b)	46.39	16.54	−1.49	8.20E−11
NF-kappa-B inhibitor alpha (Nfkbia)	38.08	16.10	−1.24	8.49E−29
Complement C1q subcomponent subunit C (C1qc)	29.82	14.74	−1.02	2.57E−11
CCAAT/enhancer-binding protein alpha (Cebpa)	39.76	12.49	−1.67	2.30E−79
Stromal cell-derived factor 1 (Cxcl12)	23.58	11.21	−1.07	2.72E−21
C-C motif chemokine 21a (Ccl21a)	64.52	9.09	−2.83	6.67E−71
C-X-C motif chemokine 13 (Cxcl13)	30.87	8.38	−1.88	1.49E−28
Sorting nexin-2 (Snx2)	15.47	6.35	−1.28	5.23E−17
Cathepsin S (Ctss)	12.32	5.89	−1.06	3.23E−07
Complement C1q subcomponent subunit B (C1qb)	13.93	5.73	−1.28	1.13E−08
Matrix metalloproteinase-23 (Mmp23)	15.41	5.72	−1.43	1.68E−12
Spondin-2 (Spon2)	13.07	5.71	−1.19	2.15E−11
Leucine-rich alpha-2-glycoprotein (Lrg1)	12.31	5.55	−1.15	7.57E−08
Peroxidasin homolog (Pxdn)	12.59	5.36	−1.23	3.23E−41

### DAE mildly increased the expression levels of DEGs participated in chondrogenesis and chondrocyte proliferation

In order to deeply investigate the effects of DAE on xiphoid cartilage, we further analyzed the expression patterns of genes participated in chondrogenesis and chondrocyte proliferation. According to the RNA-seq analysis, the expression levels of 8 chondroprogenitor markers including collagen alpha-1(II) chain (Col2a1), aggrecan core protein (Acan), hyaluronan and proteoglycan link protein 1 (Hapln1), collagen alpha-1(IX) chain (Col9a1), collagen alpha-1(XI) chain (Col11a1), transcription factor SOX-9 (Sox9), transcription factor SOX-6 (Sox6), and transcription factor SOX-5 (Sox5), and 5 proliferative chondrocyte markers including cartilage oligomeric matrix protein (Comp), fibroblast growth factor receptor 3 (Fgfr3), cartilage matrix protein (Matn1), syndecan-3 (Sdc3), and protein patched homolog 1 (Ptch1) were mildly increased under DAE treatment, as shown in Table 5.

**Table 5:** Comparison of gene expression patterns of identified genes participated in chondrogenesis and chondrocyte proliferation

Gene name	Expression level (FPKM)		log_2_ fold change (DAE/blank)	*p* value
	Blank	DAE		
*Chondroprogenitor markers*				
Collagen alpha-1(II) chain (Col2a1)	3233.52	6358.59	0.98	0
Aggrecan core protein (Acan)	694.33	1466.54	1.08	0
Hyaluronan and proteoglycan link protein 1 (Hapln1)	227.28	349.36	0.62	2.72E−114
Collagen alpha-1(IX) chain (Col9a1)	177.51	313.96	0.82	7.21E−297
Collagen alpha-1(XI) chain (Col11a1)	130.70	245.99	0.91	0
Transcription factor SOX-9 (Sox9)	36.03	64.54	0.84	2.50E−68
Transcription factor SOX-6 (Sox6)	5.21	7.61	0.55	3.88E−05
Transcription factor SOX-5 (Sox5)	3.62	5.42	0.58	1.93E−06
*Proliferative chondrocyte markers*				
Cartilage oligomeric matrix protein (Comp)	1143.78	1839.31	0.69	0
Fibroblast growth factor receptor 3 (Fgfr3)	227.52	266.94	0.23	5.04E−24
Cartilage matrix protein (Matn1)	148.54	253.96	0.77	1.19E−105
Syndecan-3 (Sdc3)	25.24	32.91	0.38	1.39E−11
Protein patched homolog 1 (Ptch1)	3.94	6.14	0.64	5.10E−06

### The results of DEGs verified by qRT-PCR were consistent with those of RNA-seq analysis

**Fig. 3:**
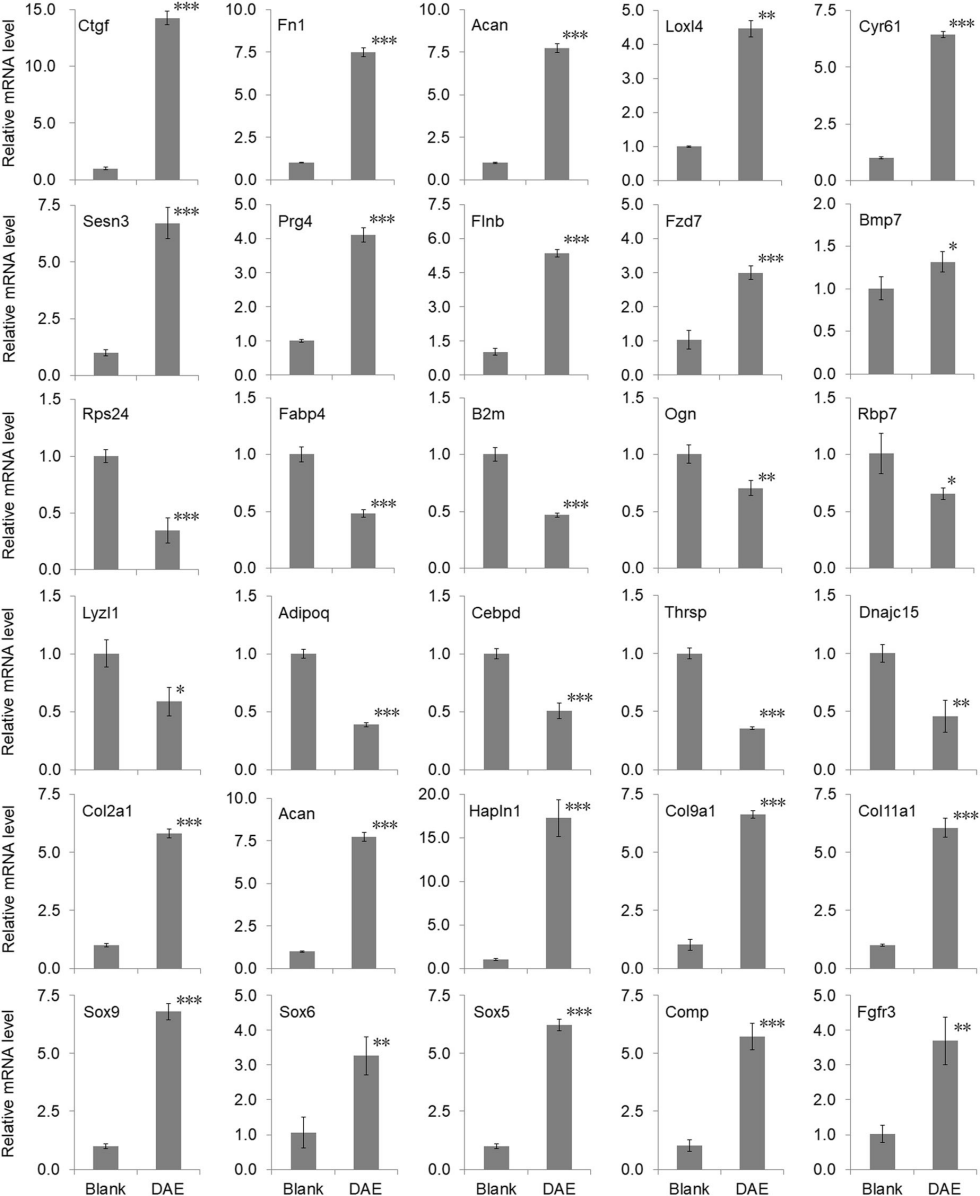
Validation of RNA-seq data by qRT-PCR. The relative expression data with their corresponding error bars were derived from three technical replicates in an experiment representative of several independent ones. The asterisk *, **, and *** indicate levels of significance of differential expression tested by Student’s *t* test with *p* value <0.05, <0.01, and <0.001, respectively. Gene expression level for each gene is calculated as the fold change of the DAE group to the blank group

Gene expression levels of 30 DEGs were verified by qRT-PCR method to corroborate the accuracy and reliability of the RNA-seq results, including 10 upregulated DGEs (Ctgf, Fn1, Acan, Loxl4, Cyr61, Sesn3, Prg4, Flnb, Fzd7, and Bmp7) involved in cartilage growth and regeneration, 10 downregulated DEGs (Rps24, Fabp4, B2m, Ogn, Rbp7, Lyzl1, Adipoq, Cebpd, Thrsp, and Dnajc15) involved in inflammation, and 10 genes (Col2a1, Acan, Hapln1, Col9a1, Col11a1, Sox9, Sox6, Sox5, Comp, and Fgfr3) involved in chondrogenesis and chondrocyte proliferation. The forward and reverse primer sequences for qRT-PCR were listed in Table 6. The relative fold change in gene expression of each gene was normalized to the internal control gene Gapdh. As shown in Fig. 3, the expression patterns of these 30 genes were consistent with the results of the RNA-seq analysis.

**Table 6:** List of genes and their specific primer sequences for qRT-PCR validation

Gene name	Primer	Sequence
Ctgf	Forward primer	CGAAGTGAGAACCGTGTGTC
	Reverse primer	CTGGCATCTCCACTCTTCCA
Fn1	Forward primer	AATGGTGACAGTTGGTTGCC
	Reverse primer	CATTGCATCGTGGTTGGCTA
Acan	Forward primer	GCTACCCTGATCCCTCATCC
	Reverse primer	GATGTCCTCTTCACCACCCA
Loxl4	Forward primer	AACAAGGGATGGGACCTGAG
	Reverse primer	ACCTTCTCCACCCAGTAAGC
Cyr61	Forward primer	TCACCCTTCTCCACTTGACC
	Reverse primer	CTGCAGTCCTCGTTGAGTTG
Sesn3	Forward primer	CTTTCCCACATGGCTGTCTG
	Reverse primer	TTGTGGTGTGAGCTTGTGTG
Prg4	Forward primer	AAAGAGACACGGAGTGCAGA
	Reverse primer	GTGGTAGTGGGAGCTGAGTT
Flnb	Forward primer	ATGCATCCCACAGTCCTTCA
	Reverse primer	CCATGACCCTCACTTCCAGT
Fzd7	Forward primer	CCTACCTAGTGGACATGCGT
	Reverse primer	CACTGCCACCATGAAGTAGC
Bmp7	Forward primer	GAAGCGTGCAAGGCATTAGA
	Reverse primer	TTCCAGAGGCAGTGTGTAGG
Rps24	Forward primer	AAACCGTCTGCTTCAGAGGA
	Reverse primer	CAAAGCCAGTTGTCTTGCCT
Fabp4	Forward primer	ATGTGCAGAAGTGGGATGGA
	Reverse primer	GTCACGCCTTTCATGACACA
B2m	Forward primer	GGACAAGGAGCCTTCTGAGT
	Reverse primer	CAACAGAAGGGCAGAAGACG
Ogn	Forward primer	GACTGTGCATCCTACGCTTC
	Reverse primer	AGTCCAGCTGAGTTTGTGGT
Rbp7	Forward primer	TTCAGGCTTTAGCTGCCAAC
	Reverse primer	CCTCGAAGTTATCGCTGCTG
Lyz1	Forward primer	AGAATCACTGCCATGTTGCC
	Reverse primer	TTCTTCCAGCCCTGCCAATA
Adipoq	Forward primer	GACAAGGCCGTTCTCTTCAC
	Reverse primer	CCCATACACTTGGAGCCAGA
Cebpd	Forward primer	AACGACCGATACCTCAGACC
	Reverse primer	TAGCTTCTCTCGCAGTCCAG
Thrsp	Forward primer	AAGGCAGTGAGGCTGAGAAT
	Reverse primer	GTGGAACTGGGCTTCTAGGT
Dnajc15	Forward primer	AGATGAGCTACGGAGACTGC
	Reverse primer	GAAGGGACGGACTATGCTGA
Col2a1	Forward primer	CAAGAAGGCCTTGCTCATCC
	Reverse primer	CAGTGTACGTGAACCTGCTG
Hapln1	Forward primer	GACAGCTACACTCCGGATCA
	Reverse primer	AGCCAAATGCTGTAGGGTCT
Col9a1	Forward primer	CCAGCACATCAAGCAGGTTT
	Reverse primer	CCTCCCAGGAAGACCAGAAG
Col11a1	Forward primer	GCAGGGAAAGAAGGTGCAAA
	Reverse primer	CTCCAGGAAGGCCTCTTTCA
Sox9	Forward primer	GAAGAATGGGCAAGCAGAGG
	Reverse primer	GCCTTGAAGATGGCGTTAGG
Sox6	Forward primer	TTTGGGCAAAGGACGAAAGG
	Reverse primer	CGGGCCTGCTCTTCATAGTA
Sox5	Forward primer	ACAGCACCTGGAGAAGTACC
	Reverse primer	ATGCGCAGTTTCTTCCCATC
Comp	Forward primer	CAGCTCAAGGCTGTCAAGTC
	Reverse primer	CTTCCAGCCCACATTTCGAG
Fgfr3	Forward primer	TGCTGGTGACTGAGGACAAT
	Reverse primer	GAGGACACCAAAGGACCAGA
Matn1	Forward primer	GGATGAGCACGTGGATTACG
	Reverse primer	ATTGCAGGTCTTTCCATCGC
Gapdh	Forward primer	CAAGGCTGAGAATGGGAAGC
	Reverse primer	GAAGACGCCAGTAGACTCCA

## Discussion

Cartilage is a specialized tissue that does not have nerves, blood vessels, or lymphatics, and has poor self-healing capacity after degeneration or injury, subsequently cause degenerative diseases, such as osteoarthritis, which affected more than 300 million people globally in 2017. Thus, cartilage regeneration currently is still a worldwide challenge [20, 21]. Among the different types of cartilage, XC can be obtained conveniently, and has a potential capacity for cartilage reconstruction due to its expansion and differentiation characteristics, and it has been widely used as a tissue model for investigation cartilage injury and repair [22–24]. Furthermore, it has been shown that XC contains cartilaginous tissue with a similar histological structure to that of articular cartilage, and histological and metabolic changes in murine XC are similar to those in articular cartilage after mechanical injury [25]. Deer antler serves as not only a unique model for studying cartilage regeneration and rapid growth but also a well-known traditional Chinese medicine for cartilage and bone enhancement. Our group previously demonstrated that the major active components in deer antler are aqueous proteins, which account for 70% of the freshly prepared aqueous extract (DAE). DAE dramatically facilitate chondrocyte viability and hold chondrocytes in a continuously proliferative state, while prevent chondrocytes from further maturation, differentiation, and apoptosis [11, 12]. Thus, the discovery of the regulation effect of deer antler on xiphoid cartilage would be a great benefit for finding complementary therapy to prevent or treat cartilage-related disease.

Based on the RNA-seq analysis, 892 genes were identified as DEGs, including 181 significantly upregulated genes and 711 significantly downregulated genes by comparing the blank group and DAE group. According to the GO enrichment analysis, the majority of the DEGs were primarily participated in the metabolic and developmental processes with binding and catalytic activities. These findings suggest that DAE potentially induce XC metabolic and developmental changes. According to the KEGG pathway enrichment analysis, the majority of the DEGs were mainly mapped to tight junction, PPAR, platelet activation, focal adhesion, complement and coagulation cascades, and AMPK signaling pathways. Among these enriched signaling pathways, tight junction functions as a paracellular gate to maintain organ or tissue homoeostasis by controlling diffusion on the basis of size and charge [26]. PPAR signaling consists of a group of nuclear receptor proteins, which functions as transcription factors to regulate cartilage growth and development [27]. Platelet activation serves as a key process during cartilage repair, since injured cartilage with platelet activation presents better chondral cellularity and regeneration [28]. Focal adhesion is the contact point that attaches chondrocytes to the pericellular cartilage matrix and links to intracellular organelles via cytoskeleton, and is involved in multiple cellular activities such as migration, proliferation, and gene expression [29]. Complement and coagulation cascade serves as a mediator of innate immunity, and play pivotal roles during cartilage physiological and pathological processes [30]. AMPK is a serine/threonine kinase that constitutively present in normal articular chondrocytes, and AMPK deficiency is associated with chondrocyte senescence and related diseases, such as osteoarthritis [31]. These findings suggest that DAE potentially secure XC homeostasis by controlling multiple signaling pathways involved in cartilage growth and development.

Among the significantly upregulated DEGs, 17 DEGs involved in cartilage growth and regeneration were identified, including Ctgf, Fn1, Acan, Loxl4, Cyr61, Sesn3, Prg4, Flnb, Fzd7, Bmp7, Bmp6, Golgb1, Pi15, Klf9, Gli2, Efna5, and Trps1. Ctgf, also known as cellular communication network factor 2 (Cnn2), is an essential growth factor that plays pivotal roles in regulating cartilage homeostasis, development, and regeneration [32]. Fn1 is a highly expressed extracellular matrix glycoprotein during cartilage repair and regeneration [33]. Acan, a critical proteoglycan component for cartilage structure, is indispensable not only for the formation of cartilage in development but also for the maintenance of cartilage after maturation [34]. Loxl4 is a family member of lysyl oxidases that play crucial roles in the maintenance of cartilage function and cartilage regeneration [35]. Cyr61, also known as cellular communication network factor 1 (Cnn1), has the capacity to promote cartilage regeneration by facilitating chondrocyte cloning in osteoarthritic cartilage [36]. Sesn3, a member of the sestrin family, is significantly downregulated in aging and osteoarthritic cartilage [37]. Prg4, also known as lubricin, is defined as a chondroprotective glycoprotein that is essential for cartilage homeostasis and repair [38]. Flnb, a type of cytoskeletal proteins, is universally expressed in the growth plate of cartilage and cartilaginous condensation of developing vertebrae, and absence of Flnb causes progressive amalgamation and malformation in the growth plate of postnatal vertebrae [39]. Fzd7, a receptor of Wnt signaling pathway, is required for column formation and regeneration of cartilage growth plate [40]. Bmp7, also known as osteogenic protein-1 (Op-1), serve as a cartilage anabolic factor that has the ability to induce matrix synthesis and promote repair in various models of articular cartilage degradation [41]. Bmp6 is a growth factor responsible for the maintenance of joint integrity and serves as a potential therapeutic molecule for cartilage repair [42]. Golgb1, also known as giantin, plays crucial roles in regulating chondrogenesis and the development of cartilage growth plate [43]. Pi15 is a key protease inhibitor involved in cartilage anabolism during cartilage repair [44]. Klf9, a family member of the C_2_H_2_ zinc finger transcription factors, is highly expressed in the vertebrae cartilage primordia [45]. Gli2 is also a C_2_H_2_ zinc finger transcription factors widely expressed in most types of chondrocytes, but at a lower level in hypertrophic chondrocytes [46]. Efna5, a cell surface GPI-bound ligand for Eph receptors, is highly expressed in normal cartilage [47]. Trps1 is predominantly expressed in the joints and limb growth plate cartilages, and acts as a key transcription factor involved in cartilage formation [48]. These findings suggest that DAE potentially facilitate XC growth and regeneration by controlling multiple functional genes involved in cartilage growth and regeneration.

Among the significantly upregulated DEGs, 30 DEGs involved in inflammation were identified, including Rps24, Fabp4, B2m, Ogn, Rbp7, Lyz1, Adipoq, Cebpd, Thrsp, Dnajc15, Cebpb, Cd36, Retn, Col4a1, Agt, Macrod1, Ifi27l2b, Nfkbia, C1qc, Cebpa, Cxcl12, Ccl21a, Cxcl13, Snx2, Ctss, C1qb, Mmp23, Spon2, Lrg1, and Pxdn. Among these DEGs, Fabp4, Macrod1, Ifi27l2b, C1qc, C1qb, and Mmp23 are involved in the process of inflammation [49–54]. B2m, a component of MHC class I molecules for monitoring inflammatory reaction, is highly expressed in osteoarthritis (OA) patients than controls, regardless of OA stage [55]. Lyz1, Dnajc15, Nfkbia, Cxcl12, Ccl21a, and Cxcl13 are considered to be arthritis-associated gene [56–61]. Cebpd is expressed at a relatively low level under normal physiological conditions and is upregulated by a variety of inflammatory stimuli, such as arthritis [62]. Furthermore, Rps24, Ogn, Rbp7, Adipoq, Thrsp, Cebpb, Cd36, Retn, Col4a1, Agt, Cebpa, Snx2, Ctss, Spon2, Lrg1, and Pxdn were also highly expressed in OA models compared to non-OAs [63–78]. These findings suggest that DAE potentially prevent XC from the risk of inflammation by supressing multiple functional genes involved in the process of inflammation. In consistent with the above results, the expression levels of a series of chondroprogenitor and proliferative chondrocyte markers, including Col2a1, Acan, Hapln1, Col9a1, Col11a1, Sox9, Sox6, Sox5, Comp, Fgfr3, Matn1, Sdc3, and Ptch1 were slightly increased under DAE treatment. Taken together, our findings suggest that DAE might serve as a complementary therapeutic regent for cartilage growth and regeneration.

## Conclusion

In summary, the present study demonstrated that DAE significantly increased the expression levels of DEGs involved in cartilage growth and regeneration, but decreased the expression levels of DEGs involved in inflammation, and mildly increased the expression levels of DEGs involved in chondrogenesis and chondrocyte proliferation. Thus, our findings suggest that DAE might serve as a complementary therapeutic regent for cartilage growth and regeneration to treat cartilage degenerative disease, such as osteoarthritis.

## Data Availability

The datasets used and/or analyzed during the current study are available from the corresponding author on reasonable request.

## Abbreviations

XC: Xiphoid cartilage
DAE: Deer antler extract
RNA-seq: RNA sequencing
qRT-PCR: Quantitative real-time PCR
SPF: Specific pathogen-free
SD: Sprague-Dawley
DEGs: Differentially expressed genes
Gapdh: Glyceraldehyde 3-phosphate dehydrogenase
KEGG: Kyoto Encyclopedia of Genes and Genomes
SRA: Sequence Read Archive
NCBI: National Center for Biotechnology Information
GO: Gene ontology
NR: Non-redundant
